# (2*S*,3′*S*,3a’*R*,5′*R*,7a’*R*)-5′-[(*E*)-5-(Furan-3-yl)-2-methyl­pent-1-en-1-yl]-3-hy­droxy-3′,4,7′-trimethyl-1′,2′,3′,3a’,5′,7a’-hexa­hydro-5*H*-spiro­[furan-2,4′-inden]-5-one

**DOI:** 10.1107/S2414314620015783

**Published:** 2020-12-11

**Authors:** Thomas Majer, Dieter Schollmeyer, Pierre Koch, Harald Gross

**Affiliations:** a Institute of Pharmaceutical Sciences, Department of Pharmaceutical Biology, Eberhard Karls University Tübingen, Auf der Morgenstelle 8, 72076 Tübingen, Germany; b Department of Organic Chemistry, Johannes Gutenberg University Mainz, Duesbergweg 10-14, 55099 Mainz, Germany; c Institute of Pharmaceutical Sciences, Department of Pharmaceutical and Medicinal Chemistry, Eberhard Karls University Tübingen, Auf der Morgenstelle 8, 72076 Tübingen, Germany; Goethe-Universität Frankfurt, Germany

**Keywords:** crystal structure, natural product, ircinianin

## Abstract

The title compound, ircinianin, C_25_H_32_O_4_, belongs to the sesterterpene tetronic acid compound family and was isolated from the marine sponge *Ircinia wistarii*. These chemical scaffolds are pharmacologically relevant, since they represent a new class of glycine receptor modulators. The furan ring makes a dihedral angle of 35.14 (12)° to the 4-hy­droxy-3-methyl­furan-2(*5H*)-one ring. The crystal packing is characterized by inter­molecular O—H⋯O hydrogen bonds, which generate [010] chains.

## Abstract 2

The title compound ircinianin belongs to the sesterterpene tetronic acid compound family and was isolated from the marine sponge *Ircinia wistarii*. These chemical scaffolds are pharmacologically relevant, since they represent a new class of glycine receptor modulators.

## Graphical abstract

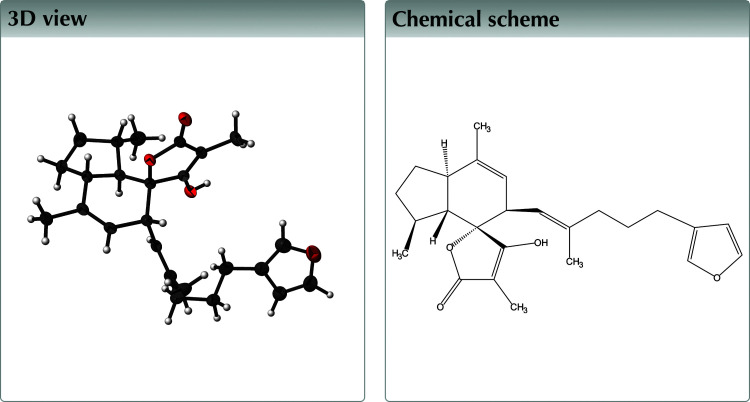

## Structure description

The genus *Ircinia* of the sea sponge family Irciniidae is a prolific source of natural products with a huge variety of different natural product classes like macrolides, alkaloids, steroids, peptides and terpenes (Coll *et al.*, 1997 ▸; Kondo *et al.*, 1992 ▸; Kobayashi *et al.*, 1995 ▸; Mau *et al.*, 1996 ▸; Chevallier *et al.*, 2006 ▸). Particularly, regarding the latter compound class, *Ircinia* spp. are known to produce unusual and rare terpenoids, especially sesterterpene tetronic acids in a linear and cyclic form, like ircinianin and its structural congeners (Hofheinz & Schönholzer, 1977 ▸; Barrow *et al.*, 1988 ▸; Coll *et al.*, 1997 ▸; Höller *et al.*, 1997 ▸; Balansa *et al.*, 2013 ▸; Balansa *et al.*, 2010 ▸).

Balansa *et al.* (2013 ▸) showed that these analogues exhibit a significant isoform-selective potentiation of glycine-gated chloride channel receptors (GlyRs). The compounds have therefore the potential to be developed either as mol­ecular tools to probe GlyR function or can serve as lead structures to treat GlyR-mediated neural disorders.

**Figure 1:**
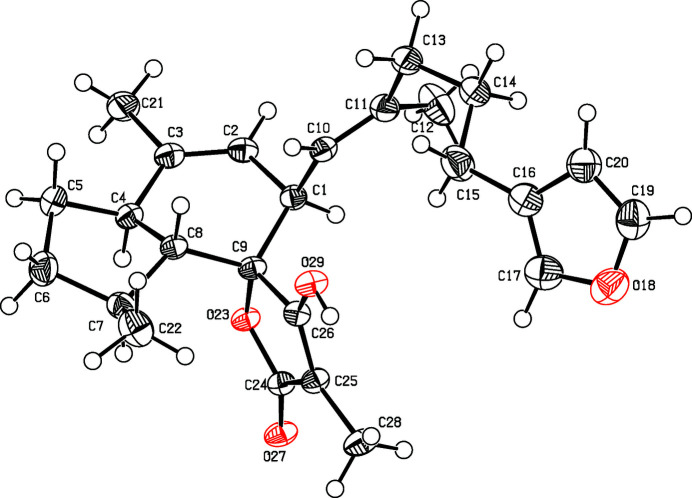
Perspective view of the title compound. Displacement ellipsoids are drawn at the 50% probability level.

**Figure 2:**
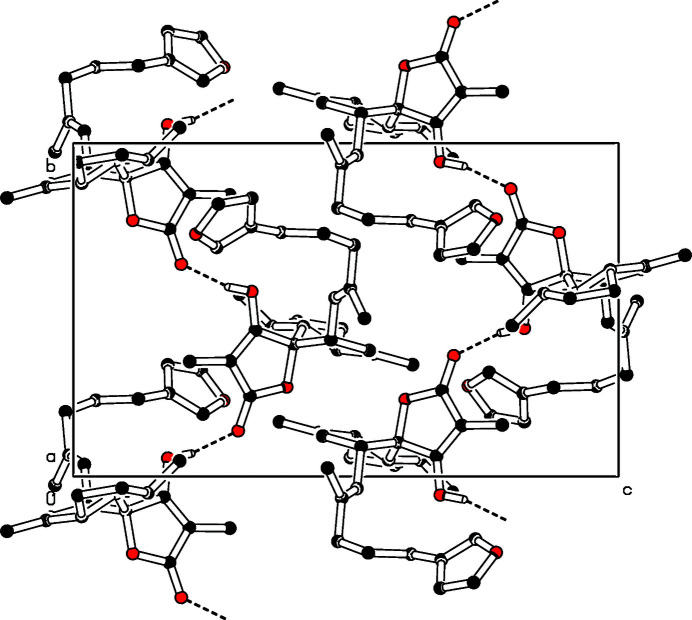
Partial packing diagram of the title compound. View along the *a*-axis.

The title compound (Fig. 1 ▸) is a polycyclic sesterterpene tetronic acid with a furan moiety. The furan ring makes a dihedral angle of 35.14 (12)° to the 4-hy­droxy-3-methyl­furan-2(*5H*)-one ring. In the crystal, the molecules are linked by O—H⋯O hydrogen bonds (Table 1 ▸, Fig. 2 ▸), forming chains parallel to the *b* axis. The crystal structure of the title compound has already been reported in 1977 by researchers from the pharmaceutical company Hoffmann La Roche (Hofheinz & Schönholzer, 1977 ▸; CCDC reference: 1180878). However, in this study the hydrogen atoms were not refined, and only the relative stereochemistry could be deduced. The absolute structure was so far solely determined by asymmetric total synthesis in 1997 (Uenishi *et al.*, 1997 ▸).

## Synthesis and crystallization

The title compound C_25_H_32_O_4_ was isolated from the marine sponge *Ircinia wistarii*. The sample (voucher number HER6) was collected from Wistarii Reef, Heron Island, Great Barrier Reef, Australia in July 1998 from a depth of 20 m. After collection, the material was stored in EtOH and kept frozen at 253 K until use.

The sponge material (800 g, wet weight) was cut into smaller pieces (2 × 2 cm) and was extracted with a solvent mixture of CHCl_3_/MeOH (1:1, *v/v*; 2 l of volume per extraction step) for three times (after 4, 8 and 20 h). The extraction solvent of each step was collected and combined. After filtration and evaporation to dryness, 25.46 g crude extract was obtained. The crude extract was redissolved in MeOH and fractioned by preparative reversed phase open column chromatography [Polygoprep 60–50 C_18_ (Macherey-Nagel) as stationary phase] using gravity and stepwise MeOH/H_2_O gradients with increasing lipophilicity and DCM. In total, eleven fractions were gained, and the ircinianin-enriched fraction (MeOH/H_2_O – 90:10) was identified by LC–MS. This fraction was then purified by reversed phase HPLC [Luna Omega 5 µm Polar C18 100 Å column, 250 × 4.6 mm, at 1.2 ml min^−1^ and UV detection at 215 nm with a 3 min gradient elution, from 20:80 to 55:45 ACN/H_2_O + 0.1% TFA, followed by ramping over 27 min to 90:10], yielding 140 mg of ircinianin, judged as pure based on total ion current profiles, ESI–MS and NMR spectrometry. Suitable crystals were prepared by slow evaporation at room temperature from a ACN/H_2_O (65:35) solution under atmospheric pressure.

Spectroscopic data of the title compound were in accordance with literature data (Balansa *et al.*, 2013 ▸). For ease of comparison with related compounds, the title compound was given in the NMR section the same numbering scheme as previously used in the literature (Balansa *et al.*, 2013 ▸):

^1^H NMR (400 MHz, MeOH-*d_4_*): δ 7.38 (H-1, *t*, 1.6), 7.26 (H-4, *m*), 6.30 (H-2, *m*), 5.11 (H-10, *dd*, 10.3, 1.1), 5.03 (H-12, *m*), 3.08 (H-11, *dm*, 10.3), 2.42 (H-15, *m*)^A^, 2.41 (H-5 *br t*, 7.5)^A^, 2.04 (H-7, *m*), 2.00 (H-17*a*, *m*), 1.89 (H-16*a*, *m*), 1.71 (H-14, *m*), 1.68 (H-6, *m*), 1.65 (H-18, *m*), 1.64 (H-25, *s*), 1.60 (H-20, *m*), 1.57 (H-9, *d*, 1.3), 1.33 (H-16*b*, *m*), 1.31 (H-17*b*, *m*), 0.92 (H-19, *d*, 6.3).

^13^C NMR (100 MHz, MeOH-*d_4_*): δ 179.2 (C-22, *s* ^C^)^B^, 177.7 (C-24, *s*)^B^, 144.0 (C-1, *d*), 140.3 (C-4, *d*), 137.1 (C-13, *s*), 136.6 (C-8, *s*), 126.5 (C-3, *s*), 125.0 (C-10, *d*), 123.6 (C-12, *d*), 112.1 (C-2, *d*), 97.5 (C-23, *s*), 86.9 (C-21, *s*), 52.0 (C-20, *d*), 48.7 (C-11, *d*), 46.2 (C-15, *d*), 40.5 (C-7, *t*), 33.6 (C-17, *t*), 33.2 (C-18, *d*), 29.5 (C-6, *t*), 27.3 (C-16, *t*), 25.3 (C-5, *t*), 20.8 (C-14, *q*), 20.7 (C-19, *q*), 16.3 (C-9, *q*), 6.1 (C-25, *q*). [^A^ Overlapping signals; ^B^ assignments inter­changeable; ^C^ implied multiplicities determined by DEPT (qC = *s*; CH = *d*; CH_2_ = *t*; CH_3_ = *q*).]

## Refinement

Crystal data, data collection and structure refinement details are summarized in Table 2 ▸. All hydrogen atoms were located in difference Fourier maps and were refined with isotropic displacement parameters.

## Supplementary Material

Crystal structure: contains datablock(s) I, global. DOI: 10.1107/S2414314620015783/bt4103sup1.cif

Structure factors: contains datablock(s) I. DOI: 10.1107/S2414314620015783/bt4103Isup2.hkl

CCDC reference: 2047802

Additional supporting information:  crystallographic information; 3D view; checkCIF report

## Figures and Tables

**Table 1:** Hydrogen-bond geometry (Å, °)

*D*—H⋯*A*	*D*—H	H⋯*A*	*D*⋯*A*	*D*—H⋯*A*
O29—H29⋯O27^i^	0.84 (3)	1.80 (3)	2.6207 (18)	166 (3)

Symmetry code: (i) 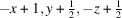 .

**Table 2:** Experimental details

Crystal data	
Chemical formula	C_25_H_32_O_4_
*M*_r_	396.50
Crystal system, space group	Orthorhombic, *P*2_1_2_1_2_1_
Temperature (K)	120
*a*, *b*, *c* (Å)	10.8217 (2), 11.1644 (2), 18.2804 (5)
*V* (Å^3^)	2208.60 (8)
*Z*	4
Radiation type	Cu *K*α
μ (mm^−1^)	0.63
Crystal size (mm)	0.91 × 0.08 × 0.08

Data collection	
Diffractometer	Stoe *IPDS* 2T
Absorption correction	Integration
*T*_min_, *T*_max_	0.914, 0.990
No. of measured, independent and observed [*I* > 2σ(*I*)] reflections	18913, 3945, 3849
*R*_int_	0.018
(sin θ/λ)_max_ (Å^−1^)	0.600

Refinement	
*R*[*F* ^2^ > 2σ(*F* ^2^)], *wR*(*F* ^2^), *S*	0.032, 0.086, 1.08
No. of reflections	3945
No. of parameters	377
H-atom treatment	All H-atom parameters refined
Δρ_max_, Δρ_min_ (e Å^−3^)	0.19, −0.20
Absolute structure	Flack *x* determined using 1639 quotients [(*I* ^+^)−(*I* ^−^)]/[(*I* ^+^)+(*I* ^−^)] (Parsons *et al.*, 2013 ▸)
Absolute structure parameter	0.03 (9)

Computer programs: *X-RED32* and *X-AREA* (Stoe & Cie, 2019 ▸), *SIR2004* (Burla *et al.*, 2005 ▸), *SHELXL2018/3* (Sheldrick, 2015 ▸) and *PLATON* (Spek, 2020 ▸).

## full crystallographic data

### Crystal data

**Table:**

C_25_H_32_O_4_	*D*_x_ = 1.192 Mg m^−^^3^
*M**_r_* = 396.50	Cu *K*α radiation, λ = 1.54186 Å
Orthorhombic, *P*2_1_2_1_2_1_	Cell parameters from 58680 reflections
*a* = 10.8217 (2) Å	θ = 2.4–68.0°
*b* = 11.1644 (2) Å	µ = 0.63 mm^−^^1^
*c* = 18.2804 (5) Å	*T* = 120 K
*V* = 2208.60 (8) Å^3^	Coloumn, colourless
*Z* = 4	0.91 × 0.08 × 0.08 mm
*F*(000) = 856	

### Data collection

**Table:**

Stoe IPDS 2T diffractometer	3945 independent reflections
Radiation source: Incoatec microSource Cu	3849 reflections with *I* > 2σ(*I*)
Detector resolution: 6.67 pixels mm^-1^	*R*_int_ = 0.018
rotation method, ω scans	θ_max_ = 67.8°, θ_min_ = 4.6°
Absorption correction: integration	*h* = −12→12
*T*_min_ = 0.914, *T*_max_ = 0.990	*k* = −13→13
18913 measured reflections	*l* = −19→21

### Refinement

**Table:**

Refinement on *F*^2^	Hydrogen site location: difference Fourier map
Least-squares matrix: full	All H-atom parameters refined
*R*[*F*^2^ > 2σ(*F*^2^)] = 0.032	*w* = 1/[σ^2^(*F*_o_^2^) + (0.0583*P*)^2^ + 0.3312*P*] where *P* = (*F*_o_^2^ + 2*F*_c_^2^)/3
*wR*(*F*^2^) = 0.086	(Δ/σ)_max_ < 0.001
*S* = 1.08	Δρ_max_ = 0.19 e Å^−^^3^
3945 reflections	Δρ_min_ = −0.20 e Å^−^^3^
377 parameters	Absolute structure: Flack *x* determined using 1639 quotients [(*I*^+^)-(*I*^-^)]/[(*I*^+^)+(*I*^-^)] (Parsons *et al.*, 2013)
0 restraints	Absolute structure parameter: 0.03 (9)
Primary atom site location: dual	

### Special details

**Table:**

Geometry. All esds (except the esd in the dihedral angle between two l.s. planes) are estimated using the full covariance matrix. The cell esds are taken into account individually in the estimation of esds in distances, angles and torsion angles; correlations between esds in cell parameters are only used when they are defined by crystal symmetry. An approximate (isotropic) treatment of cell esds is used for estimating esds involving l.s. planes.

### Fractional atomic coordinates and isotropic or equivalent isotropic displacement parameters (Å^2^)

**Table:**

	*x*	*y*	*z*	*U*_iso_*/*U*_eq_	
C1	0.49694 (18)	0.41060 (15)	0.47235 (10)	0.0219 (4)	
H1	0.428 (2)	0.357 (2)	0.4651 (13)	0.030 (6)*	
C2	0.56472 (18)	0.37841 (17)	0.54252 (10)	0.0253 (4)	
H2	0.513 (2)	0.368 (2)	0.5861 (13)	0.025 (5)*	
C3	0.68627 (19)	0.36830 (17)	0.55010 (10)	0.0259 (4)	
C4	0.76937 (17)	0.38055 (17)	0.48472 (10)	0.0236 (4)	
H4	0.786 (2)	0.301 (2)	0.4652 (13)	0.030 (6)*	
C5	0.89455 (18)	0.4432 (2)	0.49168 (11)	0.0301 (4)	
H5A	0.954 (2)	0.393 (2)	0.5169 (13)	0.029 (4)*	
H5AB	0.883 (2)	0.521 (2)	0.5200 (13)	0.029 (4)*	
C6	0.9310 (2)	0.4654 (2)	0.41093 (12)	0.0380 (5)	
H6A	0.993 (3)	0.406 (3)	0.3941 (16)	0.052 (5)*	
H6AB	0.967 (3)	0.552 (3)	0.4041 (16)	0.052 (5)*	
C7	0.81215 (17)	0.45092 (17)	0.36372 (10)	0.0259 (4)	
H7	0.815 (3)	0.373 (2)	0.3362 (15)	0.042 (7)*	
C8	0.70955 (17)	0.44944 (16)	0.42160 (10)	0.0214 (4)	
H8	0.694 (2)	0.535 (2)	0.4385 (12)	0.024 (5)*	
C9	0.58241 (17)	0.39703 (15)	0.40444 (10)	0.0205 (4)	
C10	0.44429 (17)	0.53607 (16)	0.48011 (10)	0.0235 (4)	
H10	0.500 (2)	0.602 (2)	0.4651 (13)	0.030 (6)*	
C11	0.33472 (19)	0.56473 (18)	0.50782 (10)	0.0284 (4)	
C12	0.2416 (2)	0.4744 (2)	0.53387 (18)	0.0485 (6)	
H12A	0.181 (4)	0.508 (5)	0.553 (3)	0.111 (9)*	
H12B	0.277 (5)	0.402 (4)	0.551 (3)	0.111 (9)*	
H12C	0.208 (5)	0.437 (4)	0.482 (3)	0.111 (9)*	
C13	0.2955 (2)	0.69443 (19)	0.51459 (12)	0.0333 (5)	
H13A	0.368 (3)	0.746 (3)	0.5061 (17)	0.053 (6)*	
H13B	0.260 (3)	0.702 (3)	0.5669 (18)	0.053 (6)*	
C14	0.1950 (2)	0.7288 (2)	0.45910 (12)	0.0347 (5)	
H14A	0.122 (3)	0.665 (3)	0.4642 (16)	0.054 (6)*	
H14B	0.158 (3)	0.819 (3)	0.4699 (17)	0.054 (6)*	
C15	0.2427 (2)	0.7328 (2)	0.38087 (13)	0.0426 (5)	
H15A	0.304 (3)	0.814 (3)	0.3804 (18)	0.066 (6)*	
H15B	0.280 (3)	0.652 (3)	0.3716 (18)	0.066 (6)*	
C16	0.1423 (2)	0.7499 (2)	0.32518 (12)	0.0363 (5)	
C17	0.1271 (2)	0.6877 (2)	0.26257 (13)	0.0422 (5)	
H17	0.169 (2)	0.617 (2)	0.2377 (13)	0.033 (6)*	
O18	0.02589 (17)	0.72790 (16)	0.22460 (9)	0.0467 (4)	
C19	−0.0236 (2)	0.8178 (2)	0.26580 (14)	0.0444 (6)	
H19	−0.103 (3)	0.863 (3)	0.2478 (16)	0.049 (8)*	
C20	0.0435 (2)	0.8351 (2)	0.32708 (13)	0.0404 (5)	
H20	0.029 (3)	0.894 (3)	0.3654 (16)	0.044 (7)*	
C21	0.7463 (2)	0.3359 (2)	0.62195 (12)	0.0371 (5)	
H21A	0.810 (3)	0.393 (3)	0.6351 (16)	0.052 (5)*	
H21B	0.791 (3)	0.266 (3)	0.6173 (16)	0.052 (5)*	
H21C	0.686 (3)	0.331 (3)	0.6612 (17)	0.052 (5)*	
C22	0.8012 (2)	0.5468 (2)	0.30527 (13)	0.0395 (5)	
H22A	0.727 (3)	0.533 (3)	0.2718 (17)	0.053 (4)*	
H22B	0.881 (3)	0.544 (3)	0.2759 (17)	0.053 (4)*	
H22C	0.792 (3)	0.626 (3)	0.3312 (17)	0.053 (4)*	
O23	0.59733 (12)	0.26878 (10)	0.39111 (7)	0.0211 (3)	
C24	0.55392 (17)	0.24167 (15)	0.32376 (9)	0.0217 (4)	
C25	0.50545 (18)	0.34673 (15)	0.28756 (9)	0.0228 (4)	
C26	0.52443 (16)	0.43886 (16)	0.33396 (9)	0.0209 (4)	
O27	0.56032 (13)	0.13752 (11)	0.30220 (7)	0.0271 (3)	
C28	0.4465 (2)	0.34514 (19)	0.21335 (11)	0.0321 (5)	
H28A	0.434 (4)	0.417 (4)	0.195 (2)	0.077 (6)*	
H28B	0.375 (4)	0.296 (3)	0.215 (2)	0.077 (6)*	
H28C	0.499 (4)	0.307 (3)	0.178 (2)	0.077 (6)*	
O29	0.50180 (13)	0.55511 (11)	0.32690 (7)	0.0258 (3)	
H29	0.481 (3)	0.569 (3)	0.2837 (18)	0.048 (8)*	

### Atomic displacement parameters (Å^2^)

**Table:**

	*U*^11^	*U*^22^	*U*^33^	*U*^12^	*U*^13^	*U*^23^
C1	0.0240 (8)	0.0201 (8)	0.0215 (9)	−0.0012 (7)	0.0001 (7)	−0.0006 (6)
C2	0.0310 (10)	0.0253 (9)	0.0196 (8)	0.0011 (8)	0.0014 (8)	−0.0005 (7)
C3	0.0316 (10)	0.0257 (9)	0.0205 (9)	0.0010 (8)	−0.0017 (8)	−0.0013 (7)
C4	0.0266 (9)	0.0231 (9)	0.0212 (9)	0.0005 (7)	−0.0032 (7)	−0.0007 (7)
C5	0.0255 (10)	0.0339 (10)	0.0310 (10)	−0.0016 (8)	−0.0049 (8)	−0.0019 (9)
C6	0.0274 (11)	0.0511 (14)	0.0356 (11)	−0.0073 (10)	0.0008 (9)	0.0015 (10)
C7	0.0269 (9)	0.0252 (9)	0.0255 (9)	−0.0016 (7)	0.0036 (7)	−0.0008 (7)
C8	0.0256 (9)	0.0180 (8)	0.0206 (8)	−0.0008 (7)	−0.0006 (7)	−0.0005 (7)
C9	0.0273 (9)	0.0147 (8)	0.0195 (8)	0.0010 (6)	−0.0015 (7)	−0.0012 (6)
C10	0.0263 (9)	0.0218 (8)	0.0224 (8)	−0.0006 (7)	−0.0033 (7)	−0.0026 (7)
C11	0.0295 (10)	0.0281 (9)	0.0276 (9)	0.0021 (8)	−0.0004 (7)	−0.0030 (8)
C12	0.0376 (12)	0.0383 (13)	0.0695 (18)	0.0016 (10)	0.0184 (12)	0.0015 (12)
C13	0.0331 (10)	0.0309 (11)	0.0358 (11)	0.0066 (9)	−0.0017 (9)	−0.0085 (8)
C14	0.0321 (10)	0.0346 (11)	0.0374 (11)	0.0067 (9)	−0.0001 (9)	−0.0071 (9)
C15	0.0372 (12)	0.0515 (14)	0.0393 (12)	0.0121 (11)	0.0019 (10)	0.0029 (11)
C16	0.0388 (12)	0.0351 (10)	0.0351 (11)	−0.0006 (9)	0.0030 (9)	0.0030 (9)
C17	0.0490 (13)	0.0400 (12)	0.0377 (12)	−0.0029 (11)	0.0054 (10)	0.0032 (10)
O18	0.0504 (10)	0.0526 (10)	0.0371 (8)	−0.0140 (8)	−0.0016 (7)	0.0023 (7)
C19	0.0394 (13)	0.0498 (14)	0.0439 (13)	−0.0018 (10)	−0.0021 (10)	0.0103 (11)
C20	0.0414 (12)	0.0399 (12)	0.0398 (12)	0.0038 (10)	−0.0014 (10)	0.0006 (10)
C21	0.0370 (12)	0.0530 (14)	0.0214 (10)	0.0089 (11)	−0.0025 (9)	0.0007 (9)
C22	0.0391 (12)	0.0416 (12)	0.0378 (12)	−0.0005 (10)	0.0090 (9)	0.0124 (10)
O23	0.0291 (7)	0.0148 (6)	0.0192 (6)	0.0009 (5)	−0.0029 (5)	−0.0005 (4)
C24	0.0255 (9)	0.0200 (8)	0.0196 (8)	−0.0018 (7)	0.0004 (7)	−0.0013 (6)
C25	0.0275 (9)	0.0202 (9)	0.0207 (8)	0.0004 (7)	−0.0027 (7)	0.0003 (7)
C26	0.0240 (8)	0.0180 (8)	0.0208 (8)	0.0007 (7)	0.0008 (7)	0.0011 (7)
O27	0.0388 (7)	0.0178 (6)	0.0247 (6)	0.0005 (5)	−0.0051 (6)	−0.0038 (5)
C28	0.0453 (12)	0.0258 (10)	0.0251 (9)	0.0035 (9)	−0.0114 (9)	−0.0027 (8)
O29	0.0379 (7)	0.0177 (6)	0.0217 (6)	0.0035 (5)	−0.0040 (6)	0.0013 (5)

### Geometric parameters (Å, º)

**Table:**

C1—C10	1.519 (2)	C13—H13A	0.98 (3)
C1—C2	1.521 (2)	C13—H13B	1.03 (3)
C1—C9	1.555 (2)	C14—C15	1.521 (3)
C1—H1	0.96 (2)	C14—H14A	1.07 (3)
C2—C3	1.327 (3)	C14—H14B	1.10 (3)
C2—H2	0.98 (2)	C15—C16	1.502 (3)
C3—C4	1.502 (3)	C15—H15A	1.12 (3)
C3—C21	1.509 (3)	C15—H15B	1.01 (3)
C4—C5	1.530 (3)	C16—C17	1.349 (3)
C4—C8	1.530 (2)	C16—C20	1.431 (3)
C4—H4	0.97 (2)	C17—O18	1.372 (3)
C5—C6	1.548 (3)	C17—H17	1.01 (3)
C5—H5A	0.97 (2)	O18—C19	1.365 (3)
C5—H5AB	1.02 (3)	C19—C20	1.349 (3)
C6—C7	1.557 (3)	C19—H19	1.05 (3)
C6—H6A	0.99 (3)	C20—H20	0.98 (3)
C6—H6AB	1.05 (3)	C21—H21A	0.97 (3)
C7—C22	1.517 (3)	C21—H21B	0.92 (3)
C7—C8	1.534 (2)	C21—H21C	0.97 (3)
C7—H7	1.00 (3)	C22—H22A	1.02 (3)
C8—C9	1.528 (3)	C22—H22B	1.02 (3)
C8—H8	1.02 (2)	C22—H22C	1.01 (3)
C9—O23	1.4613 (19)	O23—C24	1.352 (2)
C9—C26	1.507 (2)	C24—O27	1.230 (2)
C10—C11	1.329 (3)	C24—C25	1.445 (2)
C10—H10	0.99 (3)	C25—C26	1.349 (3)
C11—C12	1.503 (3)	C25—C28	1.499 (2)
C11—C13	1.514 (3)	C26—O29	1.327 (2)
C12—H12A	0.83 (5)	C28—H28A	0.88 (4)
C12—H12B	0.95 (5)	C28—H28B	0.95 (4)
C12—H12C	1.10 (5)	C28—H28C	0.96 (4)
C13—C14	1.536 (3)	O29—H29	0.84 (3)
			
C10—C1—C2	108.67 (15)	C11—C13—C14	112.56 (17)
C10—C1—C9	112.79 (14)	C11—C13—H13A	108.7 (19)
C2—C1—C9	111.31 (15)	C14—C13—H13A	108.3 (19)
C10—C1—H1	107.0 (14)	C11—C13—H13B	105.1 (17)
C2—C1—H1	110.1 (14)	C14—C13—H13B	108.9 (17)
C9—C1—H1	106.9 (14)	H13A—C13—H13B	113 (3)
C3—C2—C1	125.88 (17)	C15—C14—C13	112.82 (19)
C3—C2—H2	117.9 (14)	C15—C14—H14A	110.7 (16)
C1—C2—H2	116.2 (14)	C13—C14—H14A	107.4 (17)
C2—C3—C4	120.16 (17)	C15—C14—H14B	105.3 (16)
C2—C3—C21	122.54 (19)	C13—C14—H14B	111.4 (16)
C4—C3—C21	117.16 (17)	H14A—C14—H14B	109 (2)
C3—C4—C5	120.37 (16)	C16—C15—C14	113.3 (2)
C3—C4—C8	113.11 (16)	C16—C15—H15A	108.9 (17)
C5—C4—C8	101.99 (15)	C14—C15—H15A	103.4 (17)
C3—C4—H4	108.6 (14)	C16—C15—H15B	107 (2)
C5—C4—H4	106.4 (14)	C14—C15—H15B	105.6 (19)
C8—C4—H4	105.2 (14)	H15A—C15—H15B	119 (3)
C4—C5—C6	102.69 (16)	C17—C16—C20	105.8 (2)
C4—C5—H5A	111.3 (14)	C17—C16—C15	126.7 (2)
C6—C5—H5A	112.2 (14)	C20—C16—C15	127.5 (2)
C4—C5—H5AB	108.8 (13)	C16—C17—O18	111.0 (2)
C6—C5—H5AB	112.4 (13)	C16—C17—H17	136.5 (14)
H5A—C5—H5AB	109.3 (19)	O18—C17—H17	112.5 (14)
C5—C6—C7	107.52 (16)	C19—O18—C17	105.96 (18)
C5—C6—H6A	111.3 (17)	C20—C19—O18	110.6 (2)
C7—C6—H6A	108.5 (18)	C20—C19—H19	129.1 (17)
C5—C6—H6AB	110.9 (16)	O18—C19—H19	120.3 (16)
C7—C6—H6AB	109.8 (16)	C19—C20—C16	106.7 (2)
H6A—C6—H6AB	109 (2)	C19—C20—H20	127.2 (17)
C22—C7—C8	115.92 (17)	C16—C20—H20	126.1 (17)
C22—C7—C6	112.42 (18)	C3—C21—H21A	111.3 (18)
C8—C7—C6	102.52 (15)	C3—C21—H21B	110.5 (19)
C22—C7—H7	104.9 (16)	H21A—C21—H21B	102 (3)
C8—C7—H7	111.4 (16)	C3—C21—H21C	111.6 (18)
C6—C7—H7	109.8 (16)	H21A—C21—H21C	109 (2)
C9—C8—C4	110.08 (15)	H21B—C21—H21C	112 (3)
C9—C8—C7	120.96 (15)	C7—C22—H22A	111.9 (17)
C4—C8—C7	102.67 (15)	C7—C22—H22B	106.6 (17)
C9—C8—H8	105.9 (12)	H22A—C22—H22B	110 (2)
C4—C8—H8	108.2 (12)	C7—C22—H22C	107.2 (18)
C7—C8—H8	108.6 (12)	H22A—C22—H22C	110 (2)
O23—C9—C26	101.95 (13)	H22B—C22—H22C	111 (3)
O23—C9—C8	108.06 (14)	C24—O23—C9	109.44 (13)
C26—C9—C8	115.58 (15)	O27—C24—O23	118.94 (16)
O23—C9—C1	107.11 (13)	O27—C24—C25	129.87 (17)
C26—C9—C1	113.87 (14)	O23—C24—C25	111.19 (14)
C8—C9—C1	109.53 (14)	C26—C25—C24	106.00 (15)
C11—C10—C1	126.34 (18)	C26—C25—C28	130.00 (17)
C11—C10—H10	118.1 (14)	C24—C25—C28	124.01 (16)
C1—C10—H10	115.5 (14)	O29—C26—C25	131.03 (16)
C10—C11—C12	123.88 (19)	O29—C26—C9	117.58 (15)
C10—C11—C13	120.76 (19)	C25—C26—C9	111.38 (15)
C12—C11—C13	115.36 (19)	C25—C28—H28A	114 (3)
C11—C12—H12A	111 (3)	C25—C28—H28B	109 (2)
C11—C12—H12B	114 (3)	H28A—C28—H28B	114 (3)
H12A—C12—H12B	125 (4)	C25—C28—H28C	111 (2)
C11—C12—H12C	102 (3)	H28A—C28—H28C	104 (3)
H12A—C12—H12C	106 (4)	H28B—C28—H28C	103 (3)
H12B—C12—H12C	95 (3)	C26—O29—H29	109 (2)
			
C10—C1—C2—C3	−109.2 (2)	C1—C10—C11—C12	−2.5 (3)
C9—C1—C2—C3	15.6 (3)	C1—C10—C11—C13	177.95 (18)
C1—C2—C3—C4	−4.4 (3)	C10—C11—C13—C14	107.9 (2)
C1—C2—C3—C21	180.00 (19)	C12—C11—C13—C14	−71.7 (3)
C2—C3—C4—C5	142.7 (2)	C11—C13—C14—C15	−69.9 (3)
C21—C3—C4—C5	−41.5 (3)	C13—C14—C15—C16	172.2 (2)
C2—C3—C4—C8	21.9 (3)	C14—C15—C16—C17	−133.3 (2)
C21—C3—C4—C8	−162.28 (18)	C14—C15—C16—C20	46.9 (3)
C3—C4—C5—C6	−165.52 (18)	C20—C16—C17—O18	0.3 (3)
C8—C4—C5—C6	−39.4 (2)	C15—C16—C17—O18	−179.6 (2)
C4—C5—C6—C7	16.7 (2)	C16—C17—O18—C19	−0.5 (3)
C5—C6—C7—C22	137.51 (19)	C17—O18—C19—C20	0.5 (3)
C5—C6—C7—C8	12.3 (2)	O18—C19—C20—C16	−0.4 (3)
C3—C4—C8—C9	−50.8 (2)	C17—C16—C20—C19	0.1 (3)
C5—C4—C8—C9	178.42 (14)	C15—C16—C20—C19	179.9 (2)
C3—C4—C8—C7	179.09 (15)	C26—C9—O23—C24	−0.34 (18)
C5—C4—C8—C7	48.36 (17)	C8—C9—O23—C24	−122.55 (15)
C22—C7—C8—C9	77.3 (2)	C1—C9—O23—C24	119.52 (15)
C6—C7—C8—C9	−159.85 (17)	C9—O23—C24—O27	178.94 (16)
C22—C7—C8—C4	−159.65 (18)	C9—O23—C24—C25	−0.9 (2)
C6—C7—C8—C4	−36.81 (18)	O27—C24—C25—C26	−177.91 (19)
C4—C8—C9—O23	−54.40 (18)	O23—C24—C25—C26	2.0 (2)
C7—C8—C9—O23	65.0 (2)	O27—C24—C25—C28	2.5 (3)
C4—C8—C9—C26	−167.82 (14)	O23—C24—C25—C28	−177.65 (18)
C7—C8—C9—C26	−48.4 (2)	C24—C25—C26—O29	177.21 (18)
C4—C8—C9—C1	61.96 (18)	C28—C25—C26—O29	−3.2 (4)
C7—C8—C9—C1	−178.59 (15)	C24—C25—C26—C9	−2.2 (2)
C10—C1—C9—O23	−163.92 (14)	C28—C25—C26—C9	177.4 (2)
C2—C1—C9—O23	73.62 (17)	O23—C9—C26—O29	−177.86 (14)
C10—C1—C9—C26	−52.0 (2)	C8—C9—C26—O29	−61.0 (2)
C2—C1—C9—C26	−174.48 (14)	C1—C9—C26—O29	67.2 (2)
C10—C1—C9—C8	79.12 (18)	O23—C9—C26—C25	1.60 (19)
C2—C1—C9—C8	−43.34 (18)	C8—C9—C26—C25	118.51 (17)
C2—C1—C10—C11	−88.2 (2)	C1—C9—C26—C25	−113.39 (17)
C9—C1—C10—C11	147.88 (19)		

### Hydrogen-bond geometry (Å, º)

**Table:**

*D*—H···*A*	*D*—H	H···*A*	*D*···*A*	*D*—H···*A*
O29—H29···O27^i^	0.84 (3)	1.80 (3)	2.6207 (18)	166 (3)

Symmetry code: (i) −*x*+1, *y*+1/2, −*z*+1/2.
